# Low body mass index negatively affects muscle mass and intramuscular fat of chronic stroke survivors

**DOI:** 10.1371/journal.pone.0211145

**Published:** 2019-01-18

**Authors:** Naoki Akazawa, Kazuhiro Harada, Naomi Okawa, Kimiyuki Tamura, Hideki Moriyama

**Affiliations:** 1 Department of Physical Therapy, Faculty of Health and Welfare, Tokushima Bunri University, Tokushima, Tokushima, Japan; 2 Department of Physical Therapy, Faculty of Health, Medical Care, and Welfare, Kibi International University, Takahashi, Okayama, Japan; 3 Department of Rehabilitation, Kasei Tamura Hospital, Wakayama, Wakayama, Japan; 4 Life and Medical Sciences Area, Health Sciences Discipline, Kobe University, Kobe, Hyogo, Japan; Beth Israel Deaconess Medical Center, UNITED STATES

## Abstract

**Objective:**

Relationship between secondary changes in skeletal muscle and body weight in chronic stroke survivors has not yet been carefully examined. The objective of this study was to clarify the relationships between muscle mass, intramuscular fat, and body weight in chronic stroke survivors.

**Methods:**

Seventy-two chronic stroke survivors participated in this study. Transverse ultrasound images were acquired using B-mode ultrasound imaging. Quadriceps muscle mass and intramuscular fat were assessed based on muscle thickness and echo intensity, respectively. We used a stepwise multiple regression analysis to identify the factors that were independently associated with the body mass index. We entered quadriceps thickness and echo intensity of the paretic and non-paretic sides into another stepwise multiple regression model to avoid multicollinearity. Age, sex, type of stroke, time since stroke, thigh length, number of medications, and an updated Charlson comorbidity index were included as the independent variables.

**Results:**

The quadriceps thickness and echo intensity of the paretic and non-paretic sides were significantly independently associated with the body mass index: quadriceps thickness of the paretic side, β = 0.52; quadriceps thickness of the non-paretic side, β = 0.55; quadriceps echo intensity of the paretic side, β = −0.35; quadriceps echo intensity of the non-paretic side, β = −0.27).

**Conclusions:**

Our results suggest that low body mass index is associated with loss of muscle mass and increased intramuscular fat on both the paretic and non-paretic sides of chronic stroke survivors. Further studies examining whether appropriate weight management, along with targeted rehabilitation programs aimed at increasing muscle mass and decreasing intramuscular fat, achieves good outcomes in chronic stroke survivors are warranted.

## Introduction

Skeletal muscle is the main effector organ of disability in stroke survivors, and chronic stroke survivors often experience secondary changes in their skeletal muscles, including decreases in muscle mass and increases in intramuscular fat [1–6]. It is accepted that these secondary changes in skeletal muscle have been caused by reinnervation, a fiber-type shift, disuse atrophy, and local inflammatory activation [7].

Less muscle mass and more intramuscular fat are associated with decreased gait independence [6], and increasing muscle mass and decreasing intramuscular fat have been shown to improve muscle strength and gait speed in chronic stroke survivors [8]. Previous studies [1, 2] have also shown a positive relationship between maximum oxygen uptake and muscle mass, and a negative relationship between insulin sensitivity and intramuscular fat content. These findings suggest that maintaining muscle mass and avoiding intramuscular fat accumulation may be one of the important managements for maintaining muscle strength, gait ability, maximum oxygen uptake, and insulin sensitivity.

Obesity has been shown to be a risk factor for cardiovascular diseases [9–12]. Conversely, the survival rate and degree of improvement in the ability to perform activities of daily living (ADL) after stroke are higher in obese stroke survivors than in those who are underweight [13–20]. This phenomenon has been termed the “obesity paradox” [20]. Furthermore, a previous study [21] reported that a decrease of body weight in convalescent older stroke survivors is related to decreased ability to perform ADL. These findings suggest that preventing weight loss may be essential for good outcomes. In support of this hypothesis is the finding that underweight chronic stroke survivors display decreased muscle mass and increased intramuscular fat [7]. However, the relationship between these secondary skeletal muscle changes and body weight in chronic stroke survivors has not yet been carefully examined. Understanding the relationship between body weight in chronic stroke survivors and secondary changes in their skeletal muscle would allow us to develop appropriate weight management and rehabilitation strategies to prevent secondary changes in skeletal muscle and improve their ability to perform ADL.

The purpose of this study was to clarify the relationships between muscle mass, intramuscular fat, and body weight in chronic stroke survivors.

## Materials and methods

### Study design and participants

This was a cross-sectional study. Seventy-two of the chronic stroke survivors who participated in the present study lived in the community. Overall, the study included all 28 and 50 chronic stroke survivors, respectively, who participated in our two recent studies [6, 22]. All participants were outpatients and had undergone physical therapy (range of motion exercises and gait training) once or twice a week (20 min/session). Inclusion criteria were >6 months latency/time since stroke and first-ever stroke. Prospective participants with a history of dementia or aphasia were excluded from the study.

### Ethical considerations

The study objectives and procedure were explained to the participant, and each participant who understood them and agreed to participate in this study provided written informed consent prior to participation. The study protocol and consent procedures were approved by the ethics committee of Kibi International University. The study was conducted in accordance with the Declaration of Helsinki guidelines.

### Outcome measures

Primary outcomes were quadriceps muscle mass and intramuscular fat on both the paretic and non-paretic sides. Loss of muscle mass with aging and disuse occurs in the quadriceps, particularly among the muscles of the upper and lower extremities [23, 24]. In addition, muscle mass and intramuscular fat of the quadriceps are related to gait independence in chronic stroke survivors [6]. Therefore, we examined quadriceps muscle mass and intramuscular fat. The other characteristics measured were age, sex, height, weight, body mass index (BMI), type of stroke, time since stroke, number of medications, comorbidities, subcutaneous fat mass of both the paretic and non-paretic thighs, thigh lengths on the paretic and non-paretic sides, function of the paretic lower extremity, and gait independence.

#### BMI measurement

The BMI was calculated by dividing body weight (kg) by height squared (m^2^). Body weight was measured using a digital scale and was recorded to the nearest 0.1kg. Height was measured with the participants standing and was recorded to the nearest 0.5cm.

#### Quadriceps muscle mass and intramuscular fat, and subcutaneous fat mass measurements

Details of the quadriceps muscle mass and intramuscular fat measurements were described previously [6, 22]. Transverse ultrasound images were acquired using B-mode ultrasound (Nanomaxx, SonoSite Japan, Tokyo, Japan) with a linear-array probe (L25n/13-6 MHz; Nanomaxx, SonoSite Japan, Tokyo, Japan). The muscle mass and intramuscular fat content of the rectus femoris and vastus intermedius were assessed based on muscle thickness and echo intensity [6, 22, 25–34], respectively. The validities of muscle mass and intramuscular fat measurements using ultrasound have been proven by recent studies using magnetic resonance imaging [28–30]. Images of the rectus femoris and vastus intermedius were obtained at 30% of the distance from the anterosuperior iliac spine to the proximal end of the patella (thigh length) [6, 22, 31–33]. While the participants lay in a supine position with their lower limbs relaxed, water-soluble transmission gel was applied to the skin surface of the thigh and the probe was pressed lightly against the skin to avoid deformation of the muscle. All ultrasound images were recorded by the same investigator. Muscle thickness was determined as the distance between the superficial adipose tissue/muscle interface and the deep muscle/muscle interface for rectus femoris, and as the distance between the superficial muscle/muscle interface and the bone/muscle interface for vastus intermedius. Echo intensity was measured in regions of interest that were selected to include as much muscle as possible while avoiding the bone and surrounding fascia [6, 22, 25–29, 31, 33]. Muscle thickness and echo intensity were determined using ImageJ 1.49 software (National Institute of Health, Bethesda, MD, USA) [6, 22, 25–29, 31, 33]. The echo intensity was determined by computer-assisted 8-bit gray-scale analysis and the mean echo intensity of the regions of interest was expressed as a value between 0 (black) and 255 (white) [6, 22, 25–29, 31, 33, 34]. Higher echo-intensity indicates greater amounts of intramuscular fat [34]. The sum of the thickness of rectus femoris and vastus intermedius was considered the quadriceps thickness. The echo intensity of the quadriceps was calculated as the mean echo intensity of the rectus femoris and vastus intermedius. It has been shown that reliabilities of the rectus femoris and vastus intermedius thickness and echo intensity measurements on the paretic and non-paretic sides in chronic stroke survivors are high [intraclass correlation coefficients (1.1) = 0.857 − 0.959] [6]. In addition to quadriceps thickness and echo intensity measurements, we also measured the subcutaneous fat thickness on the paretic and non-paretic thighs. Subcutaneous fat thickness was determined as the distance between the dermis/adipose tissue interface and the muscle/adipose tissue interface [6].

#### Measures of other characteristics

Comorbidities, function of the paretic lower extremity, and gait independence were assessed using the updated Charlson comorbidity index [35], Fugl–Meyer Assessment (FMA) lower extremity score [36], and Functional Independence Measure (FIM) gait score [37], respectively. The validity of predicting in-hospital mortality using the updated Charlson comorbidity index was confirmed in a previous study [35]. The FMA lower extremity score, which assesses movement, coordination, and reflexes, comprises 17 items (maximum score; 34) [36]. Each item is graded on a 3-point scale (0, cannot perform; 1, partially performs; 2, performs fully) [36]. FIM gait score ranges from 1 (total assistance) to 7 (complete independence), and the levels of physical assistance required for walking and independent walking are represented as FIM gait scores 1–5 and 6 or 7, respectively [37].

### Statistical analysis

All statistical analyses were performed using SPSS version 24 (IBM SPSS Japan, Tokyo, Japan). Variables were assessed for normality using the Shapiro–Wilk test. Parametric, non-parametric, and categorical data are presented as means (standard deviation), medians (interquartile range), and numbers (percentage), respectively. Relationships between the quadriceps thickness and echo intensity of the paretic and non-paretic sides, age, BMI, FIM gait score, and FMA lower extremity score were assessed using Pearson’s correlation coefficient or Spearman’s rank correlation coefficient. We used a stepwise multiple regression analysis to identify the factors that were independently associated with the BMI. We then entered the quadriceps thickness and echo intensity of the paretic and non-paretic sides in another stepwise multiple regression model to avoid multicollinearity. Age, sex, type of stroke, time since stroke, thigh lengths of the paretic and non-paretic sides, number of medications, and the updated Charlson comorbidity index were included as independent variables. We also included subcutaneous fat thicknesses of the paretic and non-paretic thighs in the echo intensity entry model because echo intensity is influenced by the thickness of the subcutaneous fat [38]. In the multiple regression analyses, male and female sex were coded as 1 and 2, respectively. Cerebral hemorrhage and cerebral infarction were also coded as 1 and 2, respectively. In addition, we calculated an effect size (f^2^) for the multiple regression analysis using the following equation: R^2^/ (1-R^2^) [39]. The statistical power of that analysis was based on f^2^, an alpha error of 0.05, the total sample size, and the number of predictor variables. G* Power version 3.1.9.2 (Heinrich-Heine-Universität, Düsseldorf, Germany) was used to calculate the statistical power. A value of p < 0.05 was considered to indicate statistical significance.

## Results

**Fig 1:**
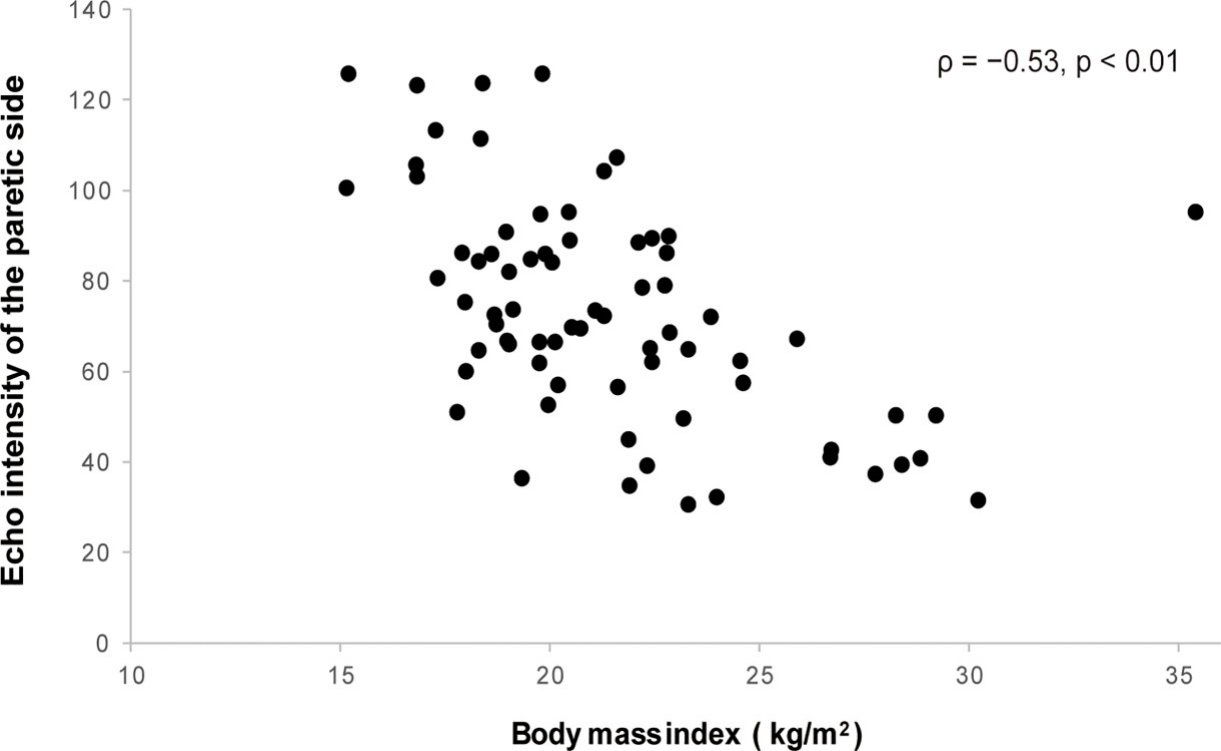
Relationship between body mass index and quadriceps echo intensity of the paretic side.

Tables 1 and 2 show the participants’ characteristics and the relationships between variables, respectively. The quadriceps thicknesses of the paretic and non-paretic sides were significantly positively associated with the BMI and the FIM gait score. The quadriceps echo intensities of the paretic and non-paretic sides were significantly negatively related to these variables (Table 2). Fig 1 shows scatter plots for BMI versus quadriceps echo intensity of the paretic side. The quadriceps thicknesses of the paretic and non-paretic sides were significantly negatively associated with the patient’s age, and the quadriceps echo intensities of the paretic and non-paretic sides were significantly positively related to the patient’s age (Table 2). There was also a significant negative relationship between the patient’s age and BMI and a significant positive relationship between the FIM gait score and the FMA lower extremity score (Table 2). The results of the stepwise multiple regression analyses are shown in Tables 3–6. There was no multicollinearity between the independent variables in the stepwise multiple regression analyses, and the variance inflation factors ranged from 1.000 to 1.473.

**Table 1:** Participant characteristics (n = 72).

Age (years)	76.0 (70.3–85.0)
Sex (male, female) [number (%)]	40 (56), 32 (44)
Height (cm)	156.6 (10.1)
Weight (kg)	52.9 (12.2)
Body mass index (kg/m^2^)	20.5 (18.8–22.9)
Type of stroke (cerebral hemorrhage, cerebral infarction) [number (%)]	26 (36), 46 (64)
Time since stroke (months)	53.2 (13.3–136.2)
Number of medications	7.0 (5.0–10.0)
Updated Charlson comorbidity Index	3.0 (2.0–4.5)
Quadriceps thickness (mm)	
Paretic side	16.6 (5.7)
Non-paretic side	18.0 (5.8)
Quadriceps echo intensity (gray-scale range 0–255)	
Paretic side	73.1 (24.4)
Non-paretic side	68.3 (23.9)
Subcutaneous fat thickness in the thigh (mm)	
Paretic thigh	5.1 (4.0–6.4)
Non-paretic thigh	4.4 (3.5–6.0)
Thigh length (mm)	
Paretic side	413.4 (28.2)
Non-paretic side	413.4 (28.2)
Fugl–Meyer Assessment lower extremity score	24.5 (19.0–29.8)
Functional independence measure gait score	4.0 (2.0–6.0)

Unless otherwise noted, the results are expressed as the median (interquartile range) or the mean (standard deviation).

**Table 2:** Relationships between quadriceps thickness and echo intensity, age, BMI, FIM gait score, and FMA lower extremity score.

	Age	BMI	FIM gait score	FMA lower extremity score	Quadriceps thickness, paretic side	Quadriceps thickness, non-paretic side	Quadriceps echo intensity, paretic side	Quadriceps echo intensity, non-paretic side
Age	1.00	−0.32**	−0.20	−0.06	−0.29*	−0.48**	0.38**	0.34**
BMI		1.00	0.16	−0.15	0.56**	0.52**	−0.53**	−0.43**
FIM gait score			1.00	0.58**	0.43**	0.40**	−0.38**	−0.28*
FMA lower extremity score				1.00	0.15	0.10	−0.14	−0.12
Quadriceps thickness, paretic side					1.00	0.80**^†^	−0.74**^†^	−0.55**^†^
Quadriceps thickness, non-paretic side						1.00	−0.72**^†^	−0.73**^†^
Quadriceps echo intensity, paretic side							1.00	0.79**^†^
Quadriceps echo intensity, non-paretic side								1.00

BMI, body mass index; FIM, functional independence measure; FMA, Fugl-Meyer Assessment

*p < 0.05

**p < 0.01

^†^Pearson’s correlation coefficient (other correlation coefficients = Spearman’s rank correlation coefficient).

**Table 3:** Stepwise multiple regression analysis (quadriceps thickness of the paretic side entry model) for body mass index.

Parameter	Partial regression coefficient	SE	95% CI of partial regression coefficient	Standardized partial regression coefficient	p
Quadriceps thickness, paretic side	0.36	0.07	0.22, 0.50	0.52	< 0.01
Type of stroke	−2.23	0.79	−3.82, −0.65	−0.29	< 0.01
Number of medications	0.24	0.11	0.03, 0.46	0.24	0.03

SE, standard error; CI, confidence interval

**Table 4:** Stepwise multiple regression analysis (quadriceps thickness of the non-paretic side entry model) for body mass index.

Parameter	Partial regression coefficient	SE	95% CI of partial regression coefficient	Standardized partial regression coefficient	p
Quadriceps thickness, non-paretic side	0.37	0.07	0.23, 0.51	0.55	< 0.01
Thigh length, non-paretic side	−0.31	0.14	−0.58, −0.03	−0.23	0.03
Type of stroke	−1.58	0.79	−3.16, −0.01	−0.21	0.04

**Table 5:** Stepwise multiple regression analysis (quadriceps echo intensity of the paretic side entry model) for body mass index.

Parameter	Partial regression coefficient	SE	95% CI of partial regression coefficient	Standardized partial regression coefficient	p
Subcutaneous fat thickness, paretic thigh	0.58	0.18	0.22, 0.93	0.36	< 0.01
Quadriceps echo intensity, paretic side	−0.06	0.02	−0.09, −0.02	−0.35	< 0.01
Type of stroke	−1.83	0.79	−3.4, −0.26	−0.24	0.02

**Table 6:** Stepwise multiple regression analysis (quadriceps echo intensity of the non-paretic side entry model) for body mass index.

Parameter	Partial regression coefficient	SE	95% CI of partial regression coefficient	Standardized partial regression coefficient	p
Subcutaneous fat thickness, non-paretic thigh	0.62	0.17	0.28, 0.96	0.39	< 0.01
Quadriceps echo intensity, non-paretic side	−0.04	0.02	−0.08, −0.01	−0.27	0.02
Type of stroke	−2.23	0.79	−3.81, −0.64	−0.29	< 0.01
Number of medications	0.24	0.11	0.03, 0.45	0.23	0.03

**Fig 2:**
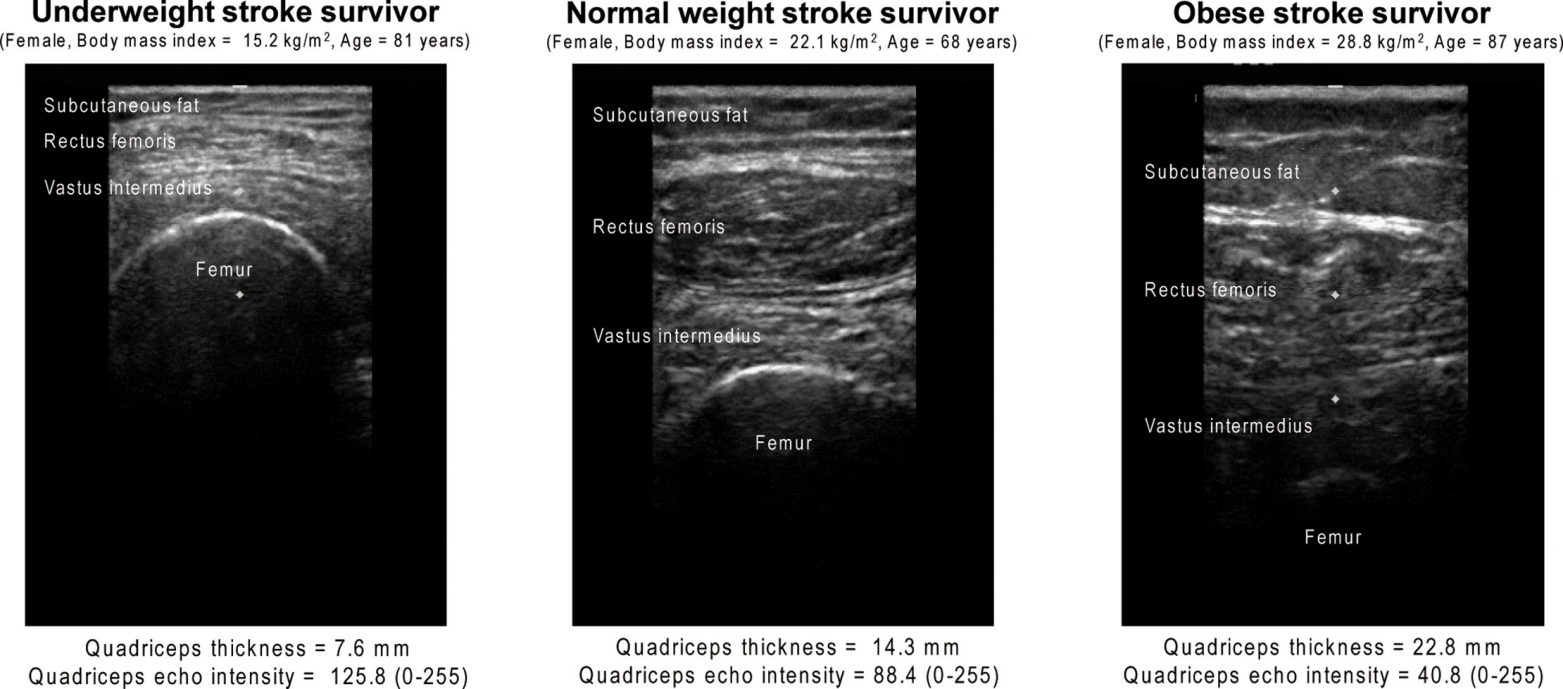
Typical ultrasound images on the paretic side of patients with different body mass indexes.

Regarding quadriceps thickness, for the paretic side entry model, the quadriceps thickness, type of stroke, and number of medications were significantly independently associated with the BMI (R^2^ = 0.38, effect size f^2^ = 0.61, statistical power = 99.8%) (Table 3). For the non-paretic side entry model, the quadriceps thickness, thigh length, and type of stroke were significantly independently associated with the BMI (R^2^ = 0.37, effect size f^2^ = 0.59, statistical power = 99.8%) (Table 4). Regarding quadriceps echo intensity, for of the paretic side entry model, the quadriceps echo intensity, subcutaneous fat thickness of the thigh, and type of stroke were significantly independently associated with the BMI (R^2^ = 0.42, effect size f^2^ = 0.72, statistical power = 99.9%) (Table 5). For the non-paretic side entry model, the quadriceps echo intensity, subcutaneous fat thickness of the thigh, type of stroke, and number of medications were significantly independently associated with the BMI (R^2^ = 0.40, effect size f^2^ = 0.67, statistical power = 99.9%) (Table 6). All stepwise multiple regression models indicated that low BMI was related to cerebral infarction. Typical ultrasound images on the paretic side for various BMI levels are shown in Fig 2.

## Discussion

Our results suggest that low BMI is associated with loss of muscle mass and increased intramuscular fat on both the paretic and non-paretic sides of chronic stroke survivors. In contrast, high BMI is associated with increased muscle mass and decreased intramuscular fat on both the paretic and non-paretic sides of these survivors.

Less muscle mass and more intramuscular fat have been shown to be associated with decreased muscle strength, gait independence, maximum oxygen uptake, and insulin sensitivity in chronic stroke survivors [1, 2, 4, 6, 22]. Results of our correlation analyses indicated that less quadriceps muscle mass and more quadriceps intramuscular fat on both the paretic and non-paretic sides were associated with lower gait independence. Based on the aforementioned findings and the relationships between muscle mass, intramuscular fat, and body weight suggested in this study, preventing loss of body weight may be important for good outcomes in chronic stroke survivors. In addition, the negative relationship between age and BMI, which was confirmed in our correlation analysis, suggests that aging of chronic stroke survivors may lead to loss of body weight. We therefore concluded that maintaining the body weight of older chronic stroke survivors is especially important. It has also been reported that decreased body weight in convalescent older stroke survivors (their mean age at the onset of the stroke was 72.5 years) is related to decreased ability to perform ADL [21].

Aging of healthy persons leads to loss of muscle mass, increased intramuscular fat, and decreased motor function [40, 41]. This study confirmed negative relationships between age and muscle mass on the paretic and non-paretic sides and a positive relationship between age and intramuscular fat on both the paretic and non-paretic sides. Secondary changes in skeletal muscle in stroke survivors may be caused by aging (as also occurs in healthy persons) in addition to innervation, fiber-type shifts, disuse atrophy, and local inflammatory activation [7]. However, we did not observe a relationship between age and gait independence in the chronic stroke survivors. Considering that the correlation analysis indicated that the FIM gait score was significantly associated with quadriceps thickness and echo intensity on both the paretic and non-paretic sides and the FMA lower extremity score, the decreased gait independence experienced by chronic stroke survivors may be attributed to the loss of quadriceps muscle mass, increased quadriceps intramuscular fat, and decreased paretic lower extremity function not aging.

Previous studies have reported that a physical activity intervention prevents age-associated increase in intermuscular fat of the mid-thigh in sedentary older adults [42] and improves muscle mass and intermuscular fat of the lower extremity in mobility-limited (Short Physical Performance Battery ≤ 9) older adults [43]. Furthermore, the addition of nutritional supplementation (whey protein and vitamin D) leads to further reduction of intermuscular fat [43]. Based on these previous findings and our results, physical activity intervention combined with appropriate weight management through nutrition supplementation could be effective for increasing muscle mass and decreasing intramuscular fat in chronic stroke survivors. Further studies examining these causal relationships are warranted.

This study has several limitations. First, because of the cross-sectional study design, we were unable to clarify the longitudinal relationships between muscle mass, intramuscular fat, and body weight. Second, the current study did not provide information about the cognitive function. Finally, although magnetic resonance imaging and computed tomography provide more accurate measurements of muscle mass and intramuscular fat, we used ultrasound to assess these variables because it is easily accessible, quick to execute, and inexpensive [44, 45]. Additionally, the validity of ultrasound measurement of muscle mass and intramuscular fat has been recently demonstrated in studies using muscle biopsy [46, 47] and magnetic resonance imaging [28–30, 48] as gold standards.

## Conclusions

Our results suggest that low BMI is associated with loss of muscle mass and increased intramuscular fat on both the paretic and non-paretic sides of chronic stroke survivors. They also suggest that high BMI is associated with increased muscle mass and decreased intramuscular fat on both the paretic and non-paretic sides of these survivors. Further studies examining whether appropriate weight management, along with targeted rehabilitation programs aimed at increasing muscle mass and decreasing intramuscular fat, achieves good outcomes in chronic stroke survivors are warranted.

## Supporting information

S1 File The dataset for this paper. (XLSX)
